# What is the long‐term clinical significance of anti‐SARS‐CoV‐2‐specific IgG?

**DOI:** 10.1111/irv.12825

**Published:** 2020-11-01

**Authors:** Gabriela Gama Freire Alberca, Ricardo Wesley Alberca

**Affiliations:** ^1^ Laboratorio de Dermatologia e Imunodeficiencias (LIM‐56) Departamento de Dermatologia Faculdade de Medicina FMUSP Universidade de Sao Paulo Sao Paulo Brazil

To the Editor:

We read with great interest the article “Clinical significance of SARS‐CoV‐2‐specific IgG detection with a rapid antibody kit for COVID‐19 patients” by Chong et al^1^ The manuscript discusses the possible implications of severe acute respiratory syndrome coronavirus‐2 (SARS‐CoV‐2)‐specific Immunoglobulin G (IgG) production and the concomitant positivity in SARS‐CoV‐2 detection by polymerase chain reaction (PCR) in asymptomatic and symptomatic patients. This study raises some interesting questions, and we would like to humbly state a few points about the possible long‐term implications of these results.

SARS‐CoV‐2‐infected patients may or may not have symptoms. Individuals that present symptoms can range from mild to severe respiratory and systemic disease, named coronavirus disease‐2019 (COVID‐19).^2^

Patients infected with SARS‐CoV‐2 can generate high titters of SARS‐CoV‐2‐specific antibody isotypes, such as IgA, IgG, and IgM.^2^ Reports have detected IgG production between 5 and 9 days after infection.^1^, ^3^, ^4^ Nevertheless, patients with some comorbidities, such as HIV, can present a delayed immune response against SARS‐CoV‐2, with detectable anti‐SARS‐CoV‐2 immunoglobulin only after 60 days after the infection.^5^

Anti‐SARS‐CoV‐2 IgG may block and neutralize SARS‐CoV‐2 and prevent COVID‐19 development.^6^, ^7^ However, studies clearly demonstrated that the presence of anti‐SARS‐CoV‐2 IgG does not indicate viral clearence.^1^, ^8^ Nevertheless, a report by Long et al^4^ verified that moderate and severe COVID‐19 patients present a distinct IgM and IgG production course. Severe patients develop IgG seroconversion earlier in relation to moderate patients.^4^ Another report by Ko et al,^9^ identified that asymptomatic and mild patients produce less neutralizing antibodies in relation to severe COVID‐19 patients.

The ability to prevent the re‐infection of anti‐SARS‐CoV‐2 IgG produced after a natural infection by SARS‐CoV‐2 is yet to be determined. Therefore, we hypothesize that the severity of the initial infection may influence the anti‐SARS‐CoV‐2 antibody production and its viral elimination efficacy.

In addition, a few confirmed cases of re‐infection have been reported.^10^, ^11^, ^12^ The two main hypotheses would be a mutation or different SARS‐CoV‐2 strain^10^ or a reduction in the levels of anti‐SARS‐CoV‐2 antibodies.^13^

A previous report on SARS‐CoV‐1 infection verified that after the viral clearance, patients remained positive with detectable titers of anti‐SARS‐CoV‐1 IgG for a year.^14^ Chong et al^1^ verified a patient IgG‐positive over 30 days after infection; nevertheless, a recent report verified a rapid reduction in anti‐SARS‐CoV‐2 IgG in 90 days after the viral clearance.^13^

The overlap of anti‐SARS‐CoV‐2 IgG and the detection of SARS‐CoV‐2 by polymerase chain reaction in nasopharyngeal and/or oropharyngeal swab samples indicate that those individuals may be able to transmit the virus. In addition to the possible rapid decay in anti‐SARS‐CoV‐2 IgG, this could lead to re‐infection and an infectious loop in the overall population.

Another possible implication is antibody‐dependent enhancement (ADE), which could facilitate the SARS‐CoV‐2 infection and make a secondary infection worst. This mechanism of infection has been described for SARS‐CoV‐1^15^ and could represent an important factor in the development of treatments for COVID‐19.

Therefore, the clinical long‐term implications of anti‐SARS‐CoV‐2 IgG need to be further investigated, with a longitudinal investigation on antibody titers, especially in asymptomatic and mild COVID‐19 patients, to assess whether a natural infection can provide long‐term protection against SARS‐CoV‐2.

## CONFLICT OF INTEREST

The authors declare that they have no conflicts of interest.

## AUTHOR CONTRIBUTIONS

**Gabriela Gama Freire Alberca:** Conceptualization (supporting); Validation (equal); Visualization (equal); Writing‐original draft (supporting); Writing‐review & editing (supporting). **Ricardo Wesley Alberca:** Conceptualization (lead); Supervision (lead); Visualization (lead); Writing‐original draft (equal); Writing‐review & editing (equal).

## Funding information

Fundação de Amparo à Pesquisa do Estado de São Paulo (FAPESP) Grant: 19/02679‐7.

## Data Availability

The data that support the findings of this study are available in the referenced material of this article.
